# Functional and phylogenetic evidence of a bacterial origin for the first enzyme in sphingolipid biosynthesis in a phylum of eukaryotic protozoan parasites

**DOI:** 10.1074/jbc.M117.792374

**Published:** 2017-06-02

**Authors:** John G. Mina, Julie K. Thye, Amjed Q. I. Alqaisi, Louise E. Bird, Robert H. Dods, Morten K. Grøftehauge, Jackie A. Mosely, Steven Pratt, Hosam Shams-Eldin, Ralph T. Schwarz, Ehmke Pohl, Paul W. Denny

**Affiliations:** From the Departments of ‡ Biosciences and; § Chemistry, Durham University, Durham DH1 3LE, United Kingdom,; the ¶ Biology Department, College of Science, University of Baghdad, Baghdad 10071, Iraq,; the ‖ Oxford Protein Production Facility UK, Research Complex at Harwell, Rutherford Appleton Laboratory, Didcot OX11 0FA, United Kingdom, and; the ** Institut für Virologie, Zentrum für Hygiene und Infektionsbiologie, Philipps-Universität Marburg, 35043 Marburg, Germany

**Keywords:** evolution, parasite metabolism, serine palmitoyltransferase, sphingolipid, Toxoplasma gondii, Apicomplexa, small angle X-ray scattering

## Abstract

*Toxoplasma gondii* is an obligate, intracellular eukaryotic apicomplexan protozoan parasite that can cause fetal damage and abortion in both animals and humans. Sphingolipids are essential and ubiquitous components of eukaryotic membranes that are both synthesized and scavenged by the Apicomplexa. Here we report the identification, isolation, and analyses of the *Toxoplasma* serine palmitoyltransferase, an enzyme catalyzing the first and rate-limiting step in sphingolipid biosynthesis: the condensation of serine and palmitoyl-CoA. In all eukaryotes analyzed to date, serine palmitoyltransferase is a highly conserved heterodimeric enzyme complex. However, biochemical and structural analyses demonstrated the apicomplexan orthologue to be a functional, homodimeric serine palmitoyltransferase localized to the endoplasmic reticulum. Furthermore, phylogenetic studies indicated that it was evolutionarily related to the prokaryotic serine palmitoyltransferase, identified in the Sphingomonadaceae as a soluble homodimeric enzyme. Therefore this enzyme, conserved throughout the Apicomplexa, is likely to have been obtained via lateral gene transfer from a prokaryote.

## Introduction

*Toxoplasma gondii* is an obligate, intracellular protozoan parasite that is able to invade and colonize a wide variety of nucleated vertebrate cells. It is a member of the Apicomplexa, a diverse phylum including important pathogens of humans and domestic animals such as *Plasmodium* (the causative agent of malaria), *Cryptosporidium* (diarrhea), *Eimeria* (coccidiosis in poultry), and *Theileria* (East Coast fever in cattle). *Toxoplasma* has emerged as an important opportunistic pathogen, and toxoplasmosis is one of the primary opportunistic diseases in the immunocompromised, particularly AIDS patients, those receiving anti-cancer chemotherapy, and organ transplant recipients (1). *Toxoplasma* infection *in utero* is also a significant cause of spontaneous abortion in economically important domestic animals (2) and congenital defects in humans (1).

**Figure 1.**
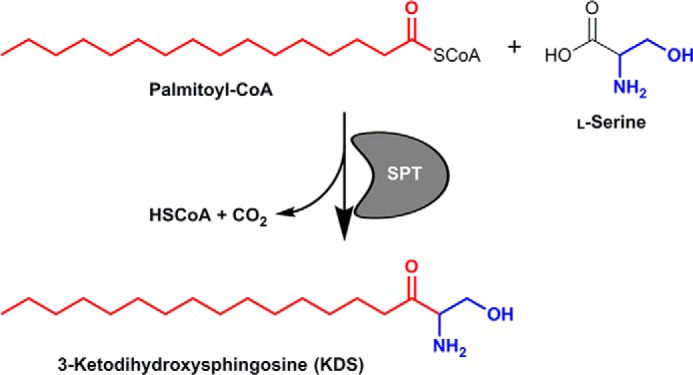
**Schematic showing the chemical reaction catalyzed by the SPT in which the enzyme catalyzes the condensation of serine and palmitoyl-CoA to form KDS with the release of coenzyme A (*HSCoA*) and CO_2_.**

As an intracellular parasite, *Toxoplasma* has a dynamic relationship with its host cell, including both the synthesis and scavenging of key lipid species (3, 4), such as sphingolipids (5–7). Sphingolipids are amphipathic lipids consisting of a sphingoid backbone acylated with a long-chain fatty acid and having a polar head group. Although the basic sphingolipid, ceramide, is a secondary signaling molecule involved in, for example, apoptosis (8–10), modified or complex sphingolipids are major components of the outer leaflet of eukaryotic plasma membranes involved, together with sterols, in the formation of microdomains commonly known as lipid rafts. These domains have been proposed to function in a diverse array of processes from the polarized trafficking of lipid-modified proteins to the assembly and activation of signal transduction complexes (11). The first, rate-limiting enzyme in sphingolipid biosynthesis is serine palmitoyltransferase (SPT),^2^ a pyridoxal phosphate (PLP)-dependent class II aminotransferase that catalyzes the Claisen-like condensation of l-serine and, typically, palmitoyl-CoA to form 3-ketodihydrosphingosine (KDS) (12) (Fig. 1). Subsequently, *N*-acylation of the sphingoid base in the endoplasmic reticulum (ER) leads to the formation of ceramide. Following transport to the Golgi apparatus, ceramide is used to form modified or complex sphingolipids, sphingomyelin (SM), or glycosphingolipid (GSL), for example (10, 13). In all eukaryotes studied to date, SPT is composed of a core heterodimer of two evolutionary related proteins that spans the membrane of the ER (14). One subunit, LCB2, contains the canonical PLP-binding and catalysis domain, whereas the other, LCB1, is not thought to bind this co-factor but to be important for complex stability (12). Both subunits are essential for enzyme activity in *Saccharomyces cerevisiae* (15), and analyses of temperature-sensitive SPT mutants have demonstrated that *de novo* synthesis of sphingolipids and their precursors is pivotal in a wide spectrum of cellular processes including endocytosis, stress responses, and protein trafficking (16–18). Members of the Prokaryota also encode a functional SPT, which was first characterized in *Sphingomonas paucimobilis* (12, 19). However, in contrast to the eukaryotic paralogue, the bacterial enzyme is a soluble, homodimeric PLP-dependent class II aminotransferase and has been proposed to represent an evolutionary precursor of the heterodimeric eukaryotic SPT (20). Despite the divergence in primary sequence, the crystal structure of the *S. paucimobilis* enzyme revealed a symmetrical dimer with the co-factor PLP bound to each subunit in a manner predicted to be conserved in the eukaryotic SPT subunit, LCB2 (21).

Like other eukaryotes, apicomplexan *Toxoplasma* and *Plasmodium* spp. synthesize sphingolipids *de novo*, including both SM and GSLs (5, 22, 23). Sphingolipid-enriched lipid microdomains have been implicated in the interaction of *Plasmodium falciparum* with the host erythrocyte (24). However, host sphingolipid biosynthesis is non-essential for the proliferation of *Toxoplasma* (6, 7), indicating that *de novo* synthesis is important for parasitism (3). *Toxoplasma* were known to produce both SM and GSLs (5), but until recently the mechanics of sphingolipid metabolism in *Toxoplasma* and other apicomplexans remained enigmatic. However, the first functionally characterized enzyme in the apicomplexan sphingolipid biosynthetic pathway has now been described as an ortholog of the yeast inositol phosphorylceramide synthase (an enzyme with no mammalian equivalent) (6). To enable further understanding and analyses, the identification and characterization of the key enzyme components in the apicomplexan *de novo* pathway is essential. Although our characterization of the *Toxoplasma* inositol phosphorylceramide synthase has initiated this process (6) significant gaps remain, not least the formal identification of the apicomplexan SPT, the first and rate-limiting step in sphingolipid biosynthesis (12). Importantly, in the absence of a defined SPT, the incorporation of tritiated serine into sphingolipid species during metabolic labeling of isolated *Toxoplasma* and *P. falciparum* indicated the presence of an active apicompexan SPT (22, 25).

Here we describe the identification and characterization of the *Toxoplasma* SPT, which represents a new class of eukaryotic enzyme with a very surprising, prokaryotic, origin. These studies shed new light on the evolution of these protozoan parasites and present a paradigm shift in the way the origin of sphingolipid biosynthesis is considered.

## Results

### A putative apicomplexan serine palmitoyltransferase

In all eukaryotes studied to date, including members of the protozoa, the first enzyme in the sphingolipid biosynthetic pathway, SPT (13), is composed of two related subunits (LCB1 and LCB2) (26). However, initial BLAST searches of the complete, annotated genome databases of both *T. gondii* (http://toxodb.org (60)) and *P. falciparum* (http://plasmodb.org (61)),^3^ using a range of LCB1 and LCB2 protein sequences, failed to locate genes encoding either SPT subunit. Given that both of these parasites have been shown to possess SPT activity (22, 25), this represented a major paradox.

**Figure 2.**
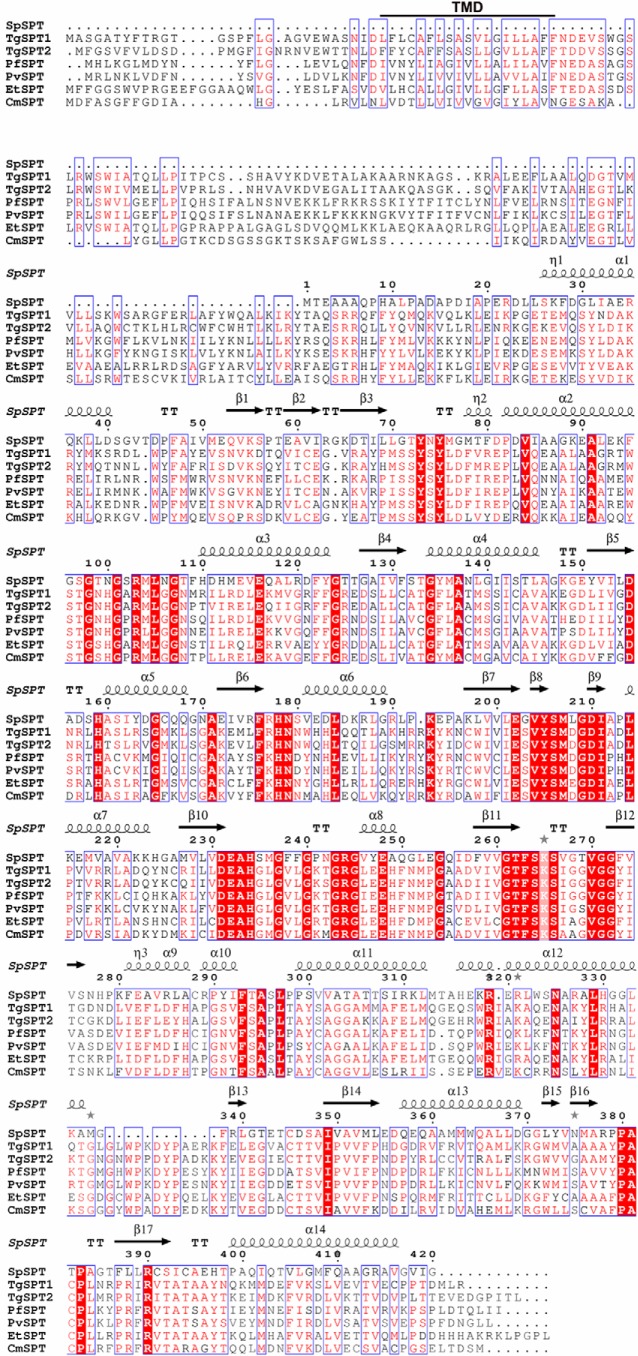
**Sequence alignment of the predicted SPT from four members of the Apicomplexa (*T. gondii*, *TgSPT1* and *TgSPT2*; *E. tenella*, *EtSPT*; *P. falciparum*, *PfSPT*; and *Plasmodium vivax*, *PvSPT*) and the characterized enzyme from the prokaryote *S. paucimobilis* (*SpSPT*).** Conserved residues (including those in the active site) identified by analyses of the SpSPT structure and homology modeling of the human functional orthologue (LCB2), are highlighted in *red*, with *red text* denoting similarity. *Blue boxes* denote conserved domains. The canonical lysine demonstrated to form an internal aldimine with the co-factor PLP at SpSPT position 265 is highlighted (21). The N-terminal extensions unique to the predicted apicomplexan enzymes harbor a transmembrane domain predicted by TMPRED (TMD, *bold* and *underlined*). The figure was produced using ESPript 3.0 (59).

Further interrogation of the *Toxoplasma* genome database using BLAST and the conserved 10 residue PLP-binding domain (PROSITE consensus PS00599) common to all eukaryotic SPT LCB2 proteins (27), identified two closely related (68% identical), tandemly encoded, predicted type II PLP-dependent aminotransferases with no known function. Surprisingly, the putative PLP-binding sites from both proteins were both completely conserved with respect to the 12 residue PLP-binding motif (GTFSKS*XXXX*GG) identified in the Sphingomonadaceae bacterial SPT (28). Further analyses demonstrated that the best characterized bacterial SPT, from *S. paucimobilis*, showed limited homology with the identified *Toxoplasma* proteins: 28 and 30% identity and 47 and 46% similarity in the C-terminal region (64% of total predicted protein) of TgSPT1 and TgSPT2, respectively (20). In addition, using the BLAST tool and the predicted *Toxoplasma* protein sequences, singly encoded orthologues of the putative apicomplexan SPT were also found in the genome databases of *Plasmodium* spp. and the chicken pathogen *Eimeria tenella.* Comparison of the primary amino acid sequences of the putative apicomplexan proteins with the bacterial SPT indicated the presence of an *N-*terminal extension, which harbors a transmembrane region absent in the prokaryotic polypeptide (Fig. 2).

Taken together, these observations clearly indicated that the putative apicomplexan SPT is radically different to those of all other eukaryotes studied thus far. To prove this, it was vital to demonstrate the functionality of the apicomplexan SPT.

### TgSPT1 is a functional serine palmitoyltransferase

**Figure 3.**
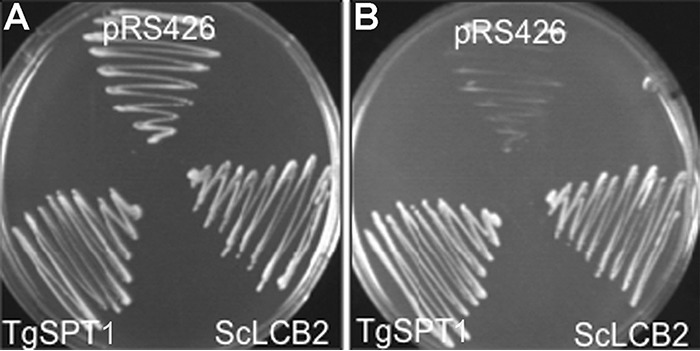
**Transformed auxotrophic yeast grown on selective medium with either galactose (*A*) or glucose (*B*).** Both ScLcb2 and TgSPT1 rescue the mutant *S. cerevisiae* that are deficient in endogenous ScLcb2 when grown in the presence of glucose (*B*). pRS246 is the empty vector control.

The complete open reading frame of the predominant, tachyzoite expressed, *Toxoplasma* SPT, TgSPT1 (see http://toxodb.org for transcriptomic data),^3^ was cloned into the yeast expression vector, pRS426-MET, to create pRS426-TgSPT1. In the auxotrophic yeast strain YPH499-HIS-GAL-LCB2, the essential PLP-binding, catalytic SPT subunit LCB2 (15) is under the control of a GAL1 promoter. In non-permissive glucose-containing SD medium, which inhibits expression from the GAL1 promoter, the yeast are non-viable. Transformation with pRS426-TgSPT1 allowed the growth of YPH499-HIS-GAL-LCB2 in this media, as did the ectopic expression of *S. cerevisiae* LCB2. In contrast, the empty vector, pRS426-MET, did not rescue the growth of the auxotrophic yeast strain (Fig. 3). These data strongly indicate that TgSPT1 is a functional orthologue of the *S. cerevisiae* LCB2 and, therefore, at least part of the *T. gondii* serine palmitoyltransferase.

**Figure 4.**
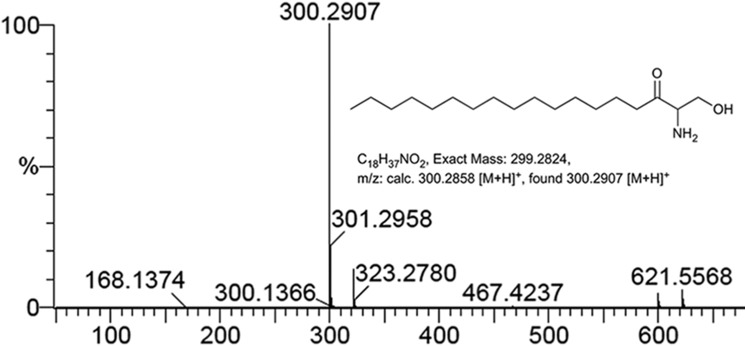
**Mass spectrometry positive ion spectrum of lipids extracted from *in vitro* reaction of TgSPT1 Δ158 with serine and palmitoyl CoA as substrates.** The peak 300.29 corresponds to the mass of 3-ketodihydrosphinganine.

To analyze the functionality of TgSPT1 *in vitro*, a series of constructs were made in collaboration with the Oxford Protein Purification Facility in the vector pOPINS3C, where the insert is N-terminally fused to a cleavable N-HIS SUMO tag (29, 30). Following triage based on expression levels and product solubility, a series of these fusion proteins (with N-terminal deletions of 143, 158, 176, and 180 amino acids) were expressed, purified, and subjected to preliminary functional analyses using palmitoyl-CoA and ^14^C-labeled serine as substrates (supplemental Fig. S1). The truncated construct TgSPT1 Δ158 was selected for further analyses. Mass spectrometry demonstrated the reaction product of the enzyme to be KDS (Fig. 4), and therefore TgSPT1 is a *bona fide* SPT.

**Figure 5.**
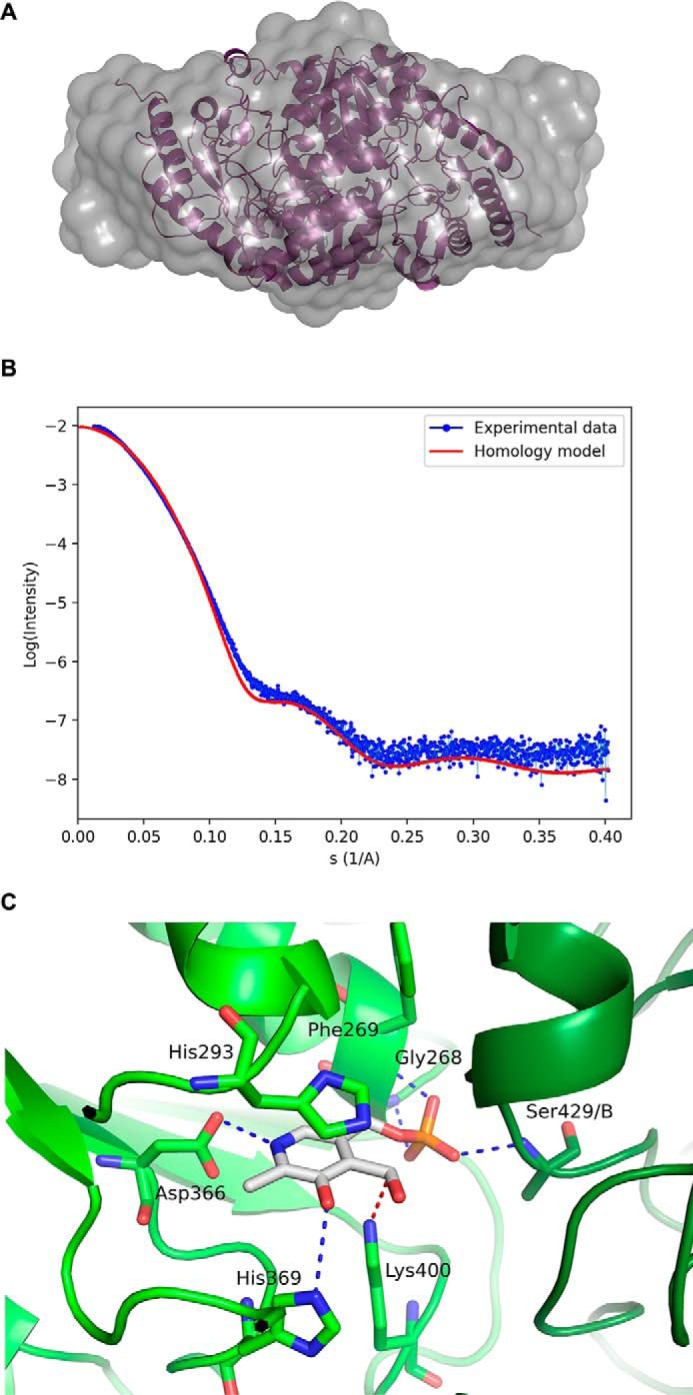
*A*, SAXS generated envelope overlaying a homology model of the TgSPT1 dimer. *B*, SAXS data (binned mode as *blue dots*), superimposed with the calculated scattering curve using the homology mode (in *red*). *C*, close-up of the PLP binding site of the homology model of TgSPT1 based on the crystal structure of SPT from *S. multivorum*. The key PLP binding residues depicted with *cyan* bonds in a ball-and-stick representation are conserved in the family (see Fig. 2). The numbering corresponds to the TgSPT1 sequence. Note that Ser-429/B belongs to the second subunit of the homodimer.

**Figure 6.**
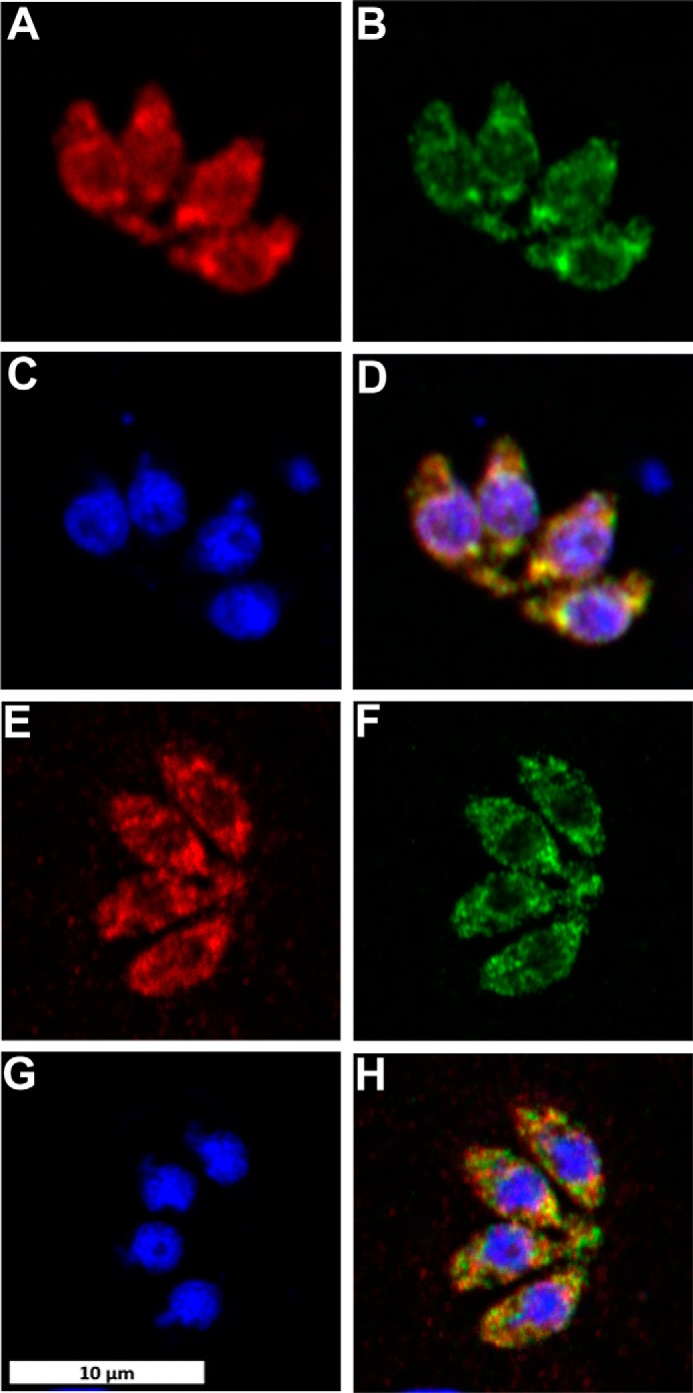
**Toxoplasma stained for ectopically expressed TgSPT1-TY (*A*, AlexaFluor594, *red*) and endogenous TgSPT1 (*E*, AlexaFluor594, *red*); ectopically expressed ER marker GFP-HDEL (*B* and *F*, AlexaFluor488, *green*); and DNA (*C* and *G*, DAPI, *blue*).** Co-localization of TgSPT1, ectopically expressed and endogenous, with GFP-HDEL is shown in merge of *A* and *B* (*D*, *yellow*) and *E* and *F* (*H*, *yellow*), respectively. The *scale bar* is equivalent to 10 μm.

**Figure 7.**
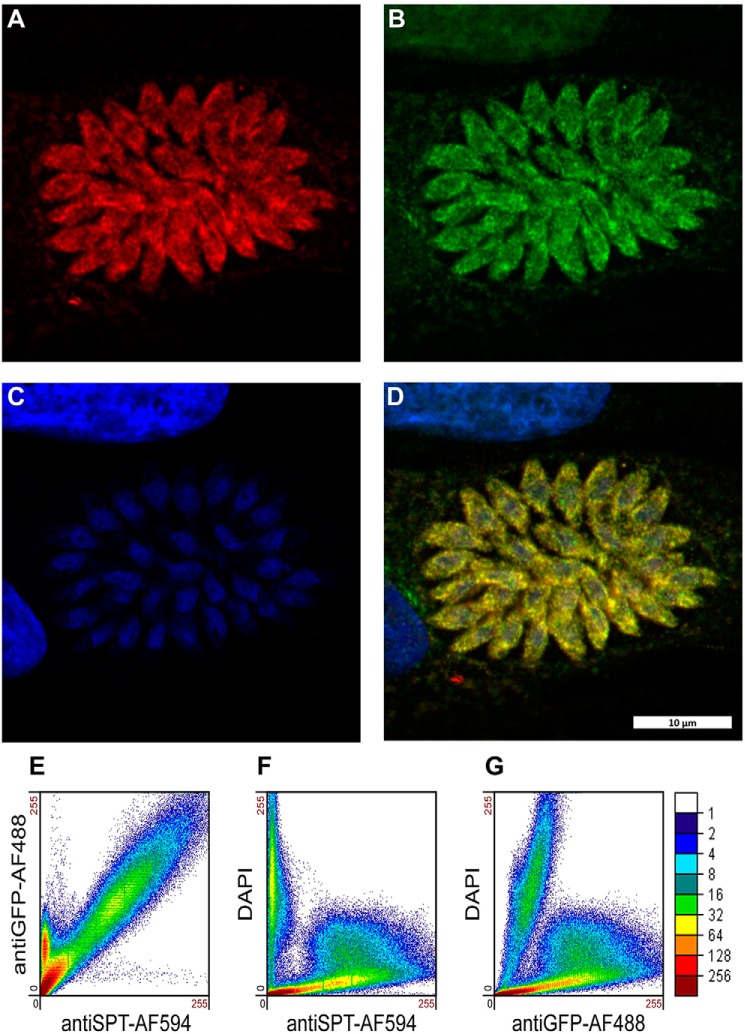
**Endogenous TgSPT1 (*A*, AlexaFluor594, *red*) and ectopically expressed ER marker GFP-HDEL (*B*, AlexaFluor488, *green*) co-localize as shown in the merged image (*D*, *yellow*).** The *scale bar* is equivalent to 10 μm. In support of this, the scatterplot (*E*) demonstrates the strong correlation of TgSPT1 (antiSPT-AF594) with GFP-HDEL (antiGFP-AF488). In contrast, neither TgSPT1 (antiSPT-AF594) nor GFP-HDEL (antiGFP-AF488) show any significant correlation with DAPI-stained (*C*) nuclei (*F* and *G*). The *color scale* represents the number of pixels as indicated.

To further understand enzyme function, small-angle X-ray scattering (SAXS) was utilized to determine the shape of the protein in solution and investigate whether TgSPT1 forms a homodimer similar to the bacterial orthologue. The results are summarized in Fig. 5*A*, which shows the experimentally derived shape of the molecule in gray as a bead model and superimposed a ribbon diagram of the homodimeric homology model of *Tg*SPT1 based on the known crystal structure of the *Sphingobacterium multivorum* SPT (52). The *ab initio* envelope shows very good agreement with the homodimeric model, where the core of the enzyme adopts a similar conformation to the bacterial orthologue. The elongated shape of the envelope indicated increased conformational flexibility of the termini of the protein. Using the homology model of the TgSPT1 dimer, the theoretical X-ray scattering data calculated with CRYSOL (31) revealed some discrepancies with the experimental data (Fig. 5*B*). Although the shape of the curve was similar, the low intensity values are higher in the experimental data consistent with a more elongated*/*or larger shape as shown in the *ab initio* envelope. Furthermore, the homology model indicated that the co-factor PLP can bind precisely to the predicted binding motifs in each monomer at the dimer interface of the structural model (Fig. 5*C*). Therefore, the *Toxoplasma* and by extension the apicomplexan SPTs are functional as homodimers. This resembles the bacterial situation (19) rather than the so far universal eukaryotic model of core heterodimeric modality (14). However, in contrast to the Prokaryota, where SPT is a soluble enzyme (20), the eukaryotic enzyme complex is associated with the membrane of the endoplasmic reticulum (14). As discussed above, the *N*-terminal extension contains a predicted transmembrane domain, and the data indicate that this does not influence functionality *in vitro*. It is noteworthy that Uniprot (www.uniprot.org) has predicted the *P. falciparum* SPT N-terminal region to target the protein to the apicoplast (32), a vestigial plastid that harbors the machinery for several lipid biosynthetic pathways (33). However, the ability of TgSPT1 to complement for a deficiency of LCB2 in auxotrophic mutant yeast (Fig. 3) indicated that the protozoal enzyme is targeted to the ER, which is the locale for SPT activity in this and other eukaryotes (14). Episomal expression of tagged TgSPT1-TY and the ER marker GFP-HDEL (34, 35) allowed co-localization by immunofluorescence microscopy and indicated that TgSPT1 is an ER rather than apicoplast-localized enzyme in *Toxoplasma* (Fig. 6, *A–D*). Furthermore, using a rat polyclonal antibody raised against TgSPT1 Δ158, the native protein was shown to have a similar ER localization pattern (Fig. 6, *E–H*). Looking at a larger vacuole showed the same localization pattern of native TgSPT1 (Fig. 7, *A–D*). In addition, the larger quantity of data available here facilitated quantitative co-localization analyses illustrated by scatterplots (Fig. 7, *E–G*). These show two-dimensional histograms of differentially labeled cell compartments (see axes labels for the channel/wavelength) at the same spatial region. A linear correlation demonstrates a strong spatial correlation between the channels, and the slope indicates the relative intensities (36, 37). The plot in Fig. 7*E* demonstrated a strong correlation of TgSPT1 (antiSPT-AF594) with GFP-HDEL (antiGFP-AF488) and ER localization. In contrast, neither TgSPT1 (antiSPT-AF594) nor GFP-HDEL (antiGFP-AF488) showed any significant correlation with DAPI-stained nuclei (Fig. 7, *F* and *G*). In an additional control experiment, TgSPT1 (antiSPT-AF594) showed no significant correlation with episomally expressed, cytosolic GFP (supplemental Fig. S2). Together, these data demonstrated that TgSPT1 has a canonical eukaryotic subcellular localization, the ER.

In summary, TgSPT1 represents a new class of eukaryotic SPTs found in the Apicomplexa. Although it functionally and structurally resembles the prokaryotic enzymes, its membrane localization and place in an apparently conventional eukaryotic synthetic pathway (6) demonstrate that it serves a conventional eukaryotic role.

### A surprising evolutionary origin for the apicomplexan serine palmitoyltransferase

**Figure 8.**
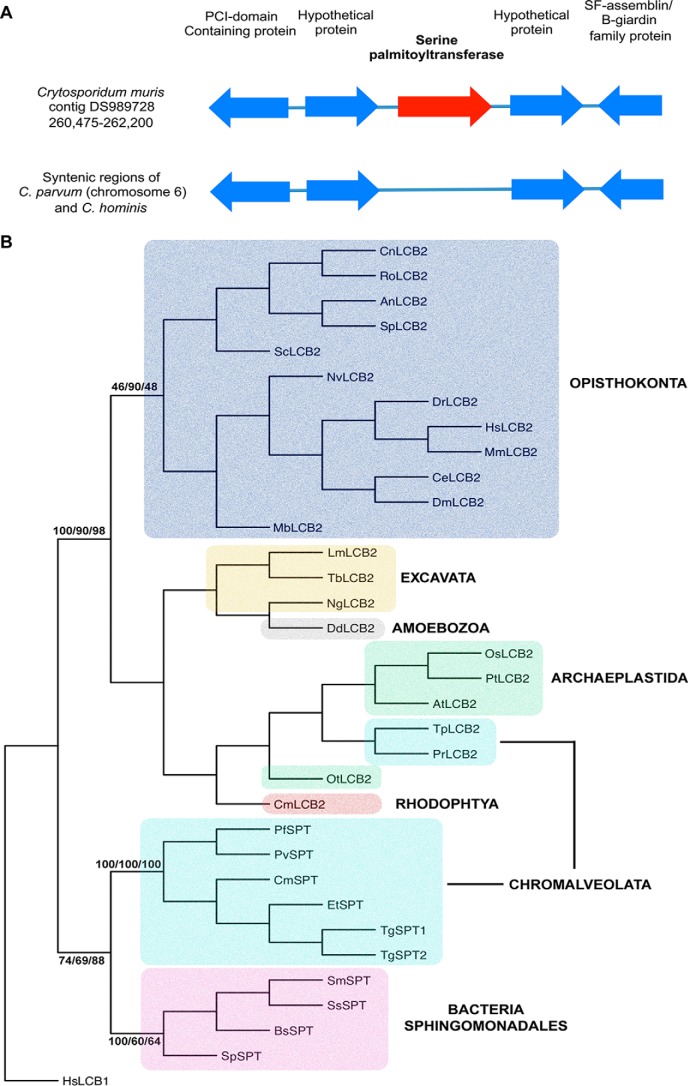
*A*, schematic illustrating the gene arrangement in the region surrounding the encoded *C. muris* serine palmitoyltransferase, compared with the syntenic regions of *C. parvum* and *C. hominis* chromosome 6. *B*, phylogenetic tree produced from a genetic distance matrix showing the relationship between the eukaryotic catalytic subunit of serine palmitoyltransferase (LCB2) and the prokaryotic and apicomplexan orthologues (SPT). The Opistokonta (animals and fungi) are colored *blue*; the Excavata (subgroup of unicellular eukaryotes) are *yellow*; Amoebozoa (amoeboid protozoa) are *gray*; Archaeplastida (plants and algae, containing cyanobacterium-derived plastid) are *green*; Rodophyta (a subgroup of the Archaeplastida, red algae) are *red*; Chromalveolata (unicellular eukaryotes containing red algal derived plastid) are *turquoise*; and Sphingomonadales (alphaproteobacteria with the ability to synthesize sphingolipids) are *pink*. The bootstrap values of the major clades are shown where they are greater than 60 at common nodes for the three methodologies employed: F-MDist, RAxML, and PhyML. The non-catalytic subunit of the human serine palmitoyltransferase (HsLCB1) was utilized as the outgroup. Sequences used in the analyses were equivalent to those aligned to TgSPT1 amino acids 228–411. Sequence information: LCB1, serine palmitoyltransferase subunit 1; LCB2, palmitoyltransferase subunit 2; SPT, serine palmitoyltransferase. NBCI accession numbers: HsLCB1: *Homo sapiens*, EAW62806; HsLCB2: *H. sapiens*, NP_004854; MmLCB2: *Mus muscalis*, NP_035609; DmLCB2: *Drosophila melanogaster*, BAA83721; CeLCB2: *Caenorhabditis elegans*, Q20375; DrLCB2, *Danio rerio*, NP_001108213; OsLCB2: *Oryza sativa*, BAD88168.1; AtLCB2: *Arabidopsis thaliana*, NP_001031932.1; DdLCB2: *Dictyostelium discodium*, XP_635115. Joint Genome Institute accession numbers: NvLCB2: *Nematostella vectensis*, 241814; MbLCB2: *Monosiga brevicollis*, 34401; PtLCB2: *Populus trichocarpa*, 834365; OtLCB2: *Ostreococcus tauri*, 16411; TpLCB2: *T. pseudonana*, 255691; PrLCB2: *P. ramorum*, 71166; NgLCB2: *Naegleria gruberi*, 82916; SpSPT: *S. paucimobilis*, Q93UV0_PSEPA; SmSPT: *S. multivorum*, A7BFV6_9SPHI; SsSPT: *Sphingobacterium spiritivorum*, A7BFV7_9SPHI; BsSPT: *Bacteriovorax stolpii*, A7BFV8_9DELT. Universal Protein Resource accession numbers: ScLCB2: *S. cerevisiae*, P40970; CnLCB2: *Cryptococcus neoformans*, J9VYF7; SpLCB2: *Schizosaccharomyces pombe*, Q09925; AnLCB2: *Aspergillus nidulans*, Q5BEC8; RoLCB2: *Rhizopus oryzae*, I1BXF5. *Cyanidioschyzon merolae* Genome Project accession number: CmLCB2: *Cyanidioschyzon merolae*, CMJ240C. ToxoDB accession numbers: TgSPT1: *T. gondii*, TGME49_090980; TgSPT2, TGME49_090970. GeneDB accession numbers: PfSPT: *P. falciparum*, PF14_0155; PvSPT, *P. vivax*; CmSPT: *C. muris*, B6ACS8_CRYMR; TbLCB2: *Trypanosoma brucei*, Tb927.10.4050; LmLCB2: *Leishmania major*, LmjF35.0320. Sanger Institute accession number: EtSPT: *E. tenella*, dev_EIMER_contig_00020813.

The data presented above detail the identification and functional characterization of TgSPT1, a eukaryotic enzyme, which in terms of its primary sequence and homodimeric structure resembles the prokaryotic “sister” enzymes. Our comprehensive sequence searches of the protozoan genome databases identified closely related orthologues of TgSPT1/2 in *Plasmodium* spp., *E. tenella*, and *Cryptosporidium muris*. Unlike *Toxoplasma*, these members of the Apicomplexa maintain a single SPT copy, indicating that TgSPT1 and TgSPT2 resulted from a gene duplication event that occurred post-speciation of the phylum. Interestingly, *Cryptosporidium hominis* and *Cryptosporidium parvum*, unlike *C. muris*, completely lack any gene encoding for SPT, despite the genomic region being syntenic between all three species (Fig. 8*A*). This suggests that *C. hominis* and *C. parvum* have selectively lost the first and rate-limiting step in sphingolipid biosynthesis, probably reflecting a specific adaptation of the parasite-host relationship.

To further analyze the evolutionary origin of the divergent apicomplexan SPT, phylogenetic analyses of a conserved region, including the PLP-binding site, were carried out. Using ClustalW (38) to align the predominant conserved region (Fig. 2 and supplemental Fig. S3), followed by Fitch Margoliash distance (F-MDist) (39), randomized accelerated maximum likelihood (RAxML) (40), and phylogeny maximum likelihood (PhyML) (41), the relationship of the apicomplexan SPT with both the eukaryotic catalytic subunit, LCB2, and the prokaryotic homodimeric SPT were determined (Fig. 8*B*). It was clear that the apicomplexan sequences do not represent conventional eukaryotic LCB2, with the kingdom to which they belong, the Chromalveolatea, split across the two major clades. The predicted catalytic subunits of the SPT from the Chromalveolate *Thalassiosira pseudonana* and *Phytophthora ramorum* group with high certainty with the conventional LCB2 subunits; however, the apicomplexan SPTs form a clade, supported by bootstrap values, with the prokaryotic sequences. This bioinformatic approach strongly indicated that the homodimeric apicomplexan enzyme is a divergent eukaryotic SPT of prokaryotic origin.

## Discussion

The *Toxoplasma* serine palmitoyltransferase, TgSPT, was identified as being encoded by two closely related genes and was found to be conserved as a single copy throughout the Apicomplexa. TgSPT1 demonstrated the ability to complement an auxotrophic yeast LCB2 mutant, and functionality was confirmed by analyses of expressed and purified TgSPT1. However, the predicted protozoan enzyme is highly divergent compared with the heterodimeric enzyme characterized throughout the Eukaryota. SAXS, coupled with homology modeling, demonstrated that the protein forms a homodimer, thereby resembling the prokaryotic rather that the eukaryotic paralogue. This relationship was further confirmed by phylogenetic analyses, which demonstrated the apicomplexan sequences as being most closely related to the prokaryotic SPT, with the protozoan SPT showing divergence from the catalytic SPT subunit (LCB2) in all other eukaryotes, including fellow members of the Chromalveolata. These data strongly indicated that the apicomplexan SPT was derived from horizontal transfer from a prokaryotic species (probably a member of Alphaproteobacteria) and demonstrated that the evolution of eukaryotic sphingolipid biosynthesis is more complex than previously recognized. These data also add to the evolutionary complexity of the Apicomplexa, protozoan parasites known to harbor a vestigial plastid (the apicoplast) as a remnant of an ancient algal endosymbiotic event (42).

## Experimental procedures

### Bioinformatics analyses

The 10 residue canonical, degenerate, PLP-binding domain common to all eukaryotic SPT subunit 2 proteins (43) was used to search the complete genome database of *T. gondii* (http://toxodb.org)^3^ with WU-BLAST (Gish, W. (1996–2003)). Two hits were identified: TGME49_090980 (TgSPT1) and TGME49_090970 (TgSPT2). The protein sequence of TgSPT1 and WU-BLAST were subsequently used to search the *Plasmodium*, *Eimeria*, and *Cryptosporidium* genome databases (http://plasmodb.org and http://genedb.org).^3^ NCBI-BLAST was used to compare the hits against the NCBI protein sequence database. Exploiting the structural data available for the bacterial *S. paucimobilis* enzyme (21), representatives of the apicomplexan and bacterial SPTs were aligned using T-Coffee Expresso (44). The resulting multiple sequence alignment was reformatted in T-Coffee with the command “t_coffee -other_pg seq_reformat -in <msa> -output sim” to yield the identity values.

### TgSPT1 isolation and cloning

*T.gondii* RH hxgprt- were propagated in vero cells (both kind gifts from Dominique Soldati-Favre, University of Geneva, Geneva, Switzerland) and isolated as described previously (45). RNA was then extracted using the RNeasy® kit (Qiagen) according to the yeast protocol. Following quantitation using Nanodrop® 2000 (ThermoFisher), cDNA was synthesized using random primers and the SuperScript® III kit (ThermoFisher) as directed by the manufacturer. Full-length TgSPT1 was then amplified by PCR using the proofreading DNA polymerase *Pfu* (Promega) and primers TgSPT5′HindIII CCC**AAGCTT**GCATGGCTTCGGGTGCAACGTACTTC and TgSPT3′NotI ATAAGAAT**GCGGCCGC**TCATCGGAGCATGTCAGTGGGTGGG (restriction sites in bold). The coding sequence was then cloned into the pET24a vector (Novagen). Subsequently, a series of deletion constructs were cloned into a series of pOPIN bacterial expression and, following transformation in a variety of *Escherichia coli* strains, screened for expression of soluble protein at the Oxford Protein Production Facility using their standardized protocols for high throughput analyses (30).

### Yeast complementation

The YPH499-HIS-GAL-LCB2 *S. cerevisiae* strain was constructed in YPH499 (Mat a; ura3-52; lys2-801amber; ade2-101ochre; trp1-63; his3-200; leu2-1) (Stratagene) by bringing the expression of the yeast LCB2 gene under the control of the stringently regulated GAL1 promoter that is repressed in the presence of glucose as described before (25, 46). The following primer sequences were used for amplification of the HIS/GAL cassette: (*a*) sequence for integration upstream of the coding region (nucleotides −200 to −150) Lcb2HisGalS, TAAGTTTCATTACTATTTTCTATTATTATCTGCAACTTTTTATTAGTTAGgggcgaattggagctccac and (*b*) sequence for integration at the initiation codon (nucleotides +1 to +50 Lcb2HisGalAS, TAAGTTTCATTACTATTTTCTATTATTATCTGCAACTTTTTATTAGTTAGgggcgaattggagctccac. The numbers indicate the nucleotide positions in the *S. cerevisiae* DNA sequence, with the adenosine of the ATG initiation codon being defined as position +1. The 19-bp sequences at the 3′ ends of these oligonucleotides that are homologous to the sequences of the vector pGAL/HIS3 and serve as a template for amplification of the GAL1/HIS3-cassette are shown with lowercase letters. Transformation into the haploid YPH499 strain, selection on minimal medium lacking histidine but containing galactose, and confirmation of the insertion of the HIS-GAL fragment were performed as previously (46). YPH499-HIS-GAL-LCB2 was maintained in SGR medium (4% galactose, 2% raffinose, 0.17% Bacto yeast nitrogen base, 0.5% ammonium sulfate) with galactose/raffinose rather than non-permissive dextrose as the carbohydrate source. For rapid cultivation of the mutant, YPGR medium (4% galactose, 2% raffinose, 1% yeast extract, 2% peptone) was routinely used.

The *S. cerevisiae* lcb2 coding region was amplified from genomic DNA (Invitrogen) using primers (Sclcb2SEcoRI GGG**GAATTC**ATGAGTACTCCTGCAAACTATACCCG and Sclcb2ASXhoI GGG**CTCGAG**AACAAAATACTTGTCGTCCTTACAATC, with restriction sites shown in bold type), and the product was cloned into pRS426MET25 to create pRS426 ScLCB2. Similarly, the TgSPT1 coding sequence was amplified (TgSPT5′SpeI **ACTAGT**ATGGCTTCGGGTGCAACGTACTTC and TgSPT3′HindIII CGC**AAGCTT**TCATCGGAGCATGTCAGTGGGTGG, with restriction sites in bold type) and cloned into the yeast expression vector to create pRS246 TgSPT1. The YPH499-HIS-GAL-LCB2 *S. cerevisiae* strain was transformed with pRS426 ScLCB2 or pRS426 TgSPT1 and functionally complemented transformants selected on non-permissive SD medium (0.17% Bacto yeast nitrogen base, 0.5% ammonium sulfate, and 2% dextrose) containing the nutritional supplements necessary to allow selection of transformants.

### TgSPT1 protein production and purification

At the Oxford Protein Purification Facility four N-terminal deletion constructs (TgSPT1 Δ143, Δ158, Δ177, and Δ180) in the pOPINS3C vector (containing a HIS-SUMO tag and a 3C protease cleavage site) showed good expression levels of soluble protein in Rosetta II (DE3) pLysS *E. coli* grown in Overnight Express^TM^ Instant TB autoinduction medium (Novagen) with 50 μg/ml ampicillin and 35 μg/ml chloramphenicol (Sigma-Aldrich). Protein production was scaled up to 2-liter baffled flasks using the same medium and conditions, incubated at 37 °C until *A*_600_ reached 0.5 then the temperature was reduced to 25 °C (TgSPT1 Δ158, Δ177, and Δ180) and incubation continued for a further 24 h or 15 °C (TgSPT1 Δ143) and 48 h. Following harvesting of the cells by centrifugation and freeze-thawing at −80 °C, the pellets were suspended in lysis buffer (50 mm Tris, pH 7.5, 500 mm NaCl, 20 mm imidazole, 0.2% Tween 20® (v/v), 10 μg/ml DNase, 10 μg/ml RNase (all Sigma-Aldrich), and EDTA-free protease inhibitors (Roche)) before lysis by sonication and isolation of the soluble fraction following centrifugation. HIS-SUMO-tagged TgSPT1 fusions were then isolated using a His trap FF 5-ml column (GE Healthcare Life Sciences) equilibrated with 50 mm Tris, pH 7.6, 500 mm NaCl, 20 mm imidazole, 25 μm PLP, and 5% glycerol (v/v) (all Sigma-Aldrich), and FPLC (Akta). Bound protein was then eluted in 50 mm Tris, pH 7.6, 500 mm NaCl, 1 m imidazole, 25 μm PLP, and 5% glycerol (v/v) before dialysis into 10 mm Tris, pH 7.6, 150 mm NaCl, 25 μm PLP, and 5% glycerol (v/v) using a Slide-A-Lyzer cassette (Thermo Scientific). To cleave the purification tag, the dialysis step was performed in the presence of the Human RhinoVirus 3C Protease (HRV 3C; Qiagen). Following concentration using a spin concentrator (Agilent Technologies), samples were injected onto on a 1-ml MonoQ 5/50 GL anion exchange column (GE Healthcare) pre-equilibrated with wash buffer (10 mm Tris, pH 8, 100 mm NaCl) using FPLC. The flow-through, containing cleaved and purified protein, was collected, concentrated, and dialyzed in appropriate buffers, and quantified using a Nanodrop® 2000.

### TgSPT enzymatic assay

Initially, TgSPT1 activity was assessed using a methodology based on a published radiochemical assay (21). In a 500-μl reaction volume (50 μm HEPES, pH 7.6, 150 mm KCl, 0.2 mm EDTA, 5% glycerol, 25 μm PLP), 20 μm of the purified protein was reacted with 20 mm l-[^14^C]serine (GE Healthcare) and 1.6 mm palmitoyl CoA (Sigma-Aldrich) for 75 min at 37 °C. The organic phase was isolated following the addition of 1 ml of CHCl_3_:CH_3_OH (2:1 v/v) and analyzed by high-performance thin layer chromatography (Merck) in a CHCl_3_:CH_3_OH:NH_4_OH, 40:10:1, solvent system. Images were captured using a AR-2000 Radio-TLC and Imaging Scanner (Bioscan). Subsequently, mass spectrometry was utilized to definitively identify the reaction products under the same conditions as above, but using cold serine and 5-fold greater volumes. Following purification as above, reaction products were analyzed, and accurate masses were obtained, using a Thermo-Finnigan LTQ FT mass spectrometer.

### Subcellular localization of TgSPT1

Primers were designed to amplify the TgSPT1 coding sequence: TgSPT5′EcoRV CGC**GATATC**ATGGCTTCGGGTGCAACGTACTTC and TgSPT3′NsiI CGC**ATGCAT**TCGGAGCATGTCAGTGGGTGG (with restriction sites in bold type). The resultant PCR product was cloned into pTUB8MycGFPPfMyoAtailTy-HX (kind gift from Dominique Soldati-Favre) (47) to create pG1-TgSPT1-TY. Transfections were carried out using a 4D Nucleofector (Lonza), protocol FI 158 and 20-μl reaction volumes in 16 reaction strips. Briefly, *Toxoplasma* were maintained in human foreskin fibroblasts (HFFs, ATCC). Parasites freshly lysed from one T75 flask of HFF cells were homogenized by passage through a 25-gauge needle and isolated by centrifugation at 1500 × *g* for 10 min at 4 °C. The pellet was resuspended in the P3 buffer with added supplement (Lonza), and *Toxoplasma* concentration was adjusted to 10^7^ ml^−1^. 20 μl of this parasite suspension was added to a dried pellet of ethanol-precipitated ∼10 μg of P30-GFP-HDEL (kind gift from Kristin Hager, University of Notre Dame) (48), and/or pG1-TgSPT1-TY plasmid was transferred to the transfection strips and electroporated. Subsequently, 100 μl of medium was added, and 10 μl or 20 μl were added to 24-well plates containing confluent HFF cells grown on glass coverslips. The plates were incubated at 37 °C, 5.0% CO_2_ for the appropriate time period.

The cells were fixed in 4% paraformaldehyde in PBS (pH 7.4) for 15 min and then permeablized with 0.4% (v/v) Triton X-100 in PBS for 10 min, before incubation in blocking buffer (PBS supplemented with 1% (w/v) BSA; Sigma-Aldrich), 0.1% fish skin gelatin (Sigma-Aldrich), and 0.1% (v/v) Triton X-100) for 15 min at room temperature. Samples were incubated with a mouse monoclonal anti-TY antibody (1:200; kind gift from Keith Gull, University of Oxford, Oxford, UK) or the primary anti-TgSPT1 Δ158 rat polyclonal (Cambridge Research Biochemicals, 1:200), and an anti-GFP rabbit polyclonal antibody (Clontech, 1:200) in blocking buffer overnight at 4 °C. After PBS washing, samples were incubated with Alexa Fluor® 594 anti-mouse or anti-rat and Alexa Fluor® 488 anti-rabbit secondary antibodies (ThermoFisher) at 1:500 in blocking buffer for 1 h at room temperature. The samples were incubated with DAPI (Sigma-Aldrich) in PBS for 10 min, mounted using Vectashield H-1000 (Vector labs), and sealed with nail polish before imaging.

All images were obtained using laser scanning confocal microscope Zeiss LSM 880 with AiryScan equipped with excitation laser 405, Argon 458, 488, 514, He-Ne 543, 594, and 633 and AiryScan filter set combinations BP 420–480 + BP 495–550, BP 420–480 + BP 495–620, BP 420–480 + LP 605, BP 465–505 + LP 525, BP 495–550 + LP 570, and BP 570–620 + LP 645. For each image, the dynamic range was checked to avoid saturation, except with the DAPI stain where host cells masked the detection of parasite nuclei at low gain/laser power values. AiryScan images were automatically processed using default values. Zeiss CZI images were exported to TIFF file format using Zen (Blue Edition version 2.3, Carl Zeiss Microscopy GmbH, 2011) and analyzed using ImageJ Fiji package (49). Co-localization was assessed using the ScatterJn plugin and scatter plots (36, 37). The scatterplots show two-dimensional histograms of two channels at the same spatial region. Data points are generated as *n*(*x*,*y*), where *n* is the number of pixels in each channel, and *x*,*y* are discrete values 0–255, and these data points are displayed as a scatterplot of 256 × 256 matrix in which the element (*x*,*y*) contains the number of data points with coordinates (*x*,*y*). The number of pixels is represented by a color scale. A linear correlation demonstrates a strong spatial correlation between the channels, and the slope indicates the relative intensities. Negative controls were checked between DAPI and the Golgi marker, pTub-GRASP-RFP (50), and no positive correlation was found.

### Homology modeling

The TgSPT1 homology model was constructed using the Δ158 SPT sequence with the HHPred server (http://toolkit.tuebingen.mg.de/hhpred (62))^3^ (51), which identified the crystal structure of SPT from *S. multivorum* (52) as the closest orthologue (Protein Data Bank code 3A2B) and aligned the sequences based on sequence identity and the predicted secondary structure of TgSPT1 and the actual secondary structure of 3A2B. The biologically relevant homodimer was used as a template to produce five preliminary models with MODELLER (53). The model with the lowest *molpdf* score was taken forward to the next step, in which PLP was added based on the known bacterial SPT structure. After another minimizing step, the two loops of residues 472–494 that are absent in the bacterial structure, were modeled using MODELLER (54). The optimal conformation was based on the lowest *molpdf* and *DOPE* scores, as well as manual inspection using interactive computer graphics.

### Small angle X-ray scattering

SAXS data were collected using the Δ158 SPT construct on Beamline B21 at the Diamond Light Source in size exclusion chromatography HPLC mode (55). Prior to data collection, the sample was concentrated to ∼5 mg/ml. The elution peak was exposed for 5 min. The images collected after the 4-min mark showed signs of radiation damage by analysis of radius of gyration and were discarded. The raw images were processed, and the background as subtracted in DAWN (56) at the beamline. Low *q* values outside the linear part of the Guinier plot were removed in ScÅtter along with *q* > 0.2519 and *D*_max_ calculated to 133 Å. Further data processing was performed using ATSAS (57). PRIMUS calculated *D*_max_ to 138 Å and the Porod volume to 150387 Å^3^, equivalent to 94 kDa using the rule of thumb of dividing the Porod volume by 1.6 Å^3^/Da, a good fit of a dimer of the TgSPT1 D158 construct at 46 kDa per monomer. Fifteen 2-fold dimer envelope models were created in DAMMIF using the ATSAS server, a consensus envelope was created by DAMAVER, and that envelope was used as a starting point for DAMMIN (58). The DAMMIN envelope was superposed with the homology model in SUPCOMB, and the result was rendered in PyMol 1.7. CRYSOL (31) was used to calculate the theoretical X-ray scattering data from the homology model.

### Phylogenetic analyses

The selected predicted protein sequences were aligned using ClustalW, edited to remove non-aligned regions, and then realigned in ClustalW with the output selected as a PHYLIP file (supplemental Fig. S3). This alignment was then subjected to three different phylogenetic analyses: F-MDist (39), RAxML (40), and PhyML (41). Bootstrap values were calculated for each analysis and used to establish the strength of the common clades in a consensus tree generated from the F-MDist data using DRAWGRAM in PHYLIP (39).

## Author contributions

J. G. M., J. K. T., and A. Q. I. A. conducted most of the experiments and analyzed the results. L. E. B. managed the construct assembly and analyses at Oxford Protein Purification Facility. R. H. D. optimized the protein expression. M. K. G. performed the SAXS experimental and analyses. J. A. M. conducted the mass spectrometry. S. P. cloned the cDNA. H. S.-E. constructed the conditional yeast mutant. R. T. S., E. P., and P. W. D. designed and managed the experimental. P. W. D. conceived the idea for the project and wrote the paper with E. P. and J. G. M.

## Supplementary Material

Supplemental Data

## Footnotes

3 Please note that the JBC is not responsible for the long-term archiving and maintenance of this site or any other third party hosted site.

## Footnotes

2 The abbreviations used are:

SPT: serine palmitoyltransferase
KDS: 3-ketodihydrosphingosine
PLP: pyridoxal phosphate
ER: endoplasmic reticulum
LCB: long-chain base
SM: sphingomyelin
GSL: glycosphingolipid
SAXS: small-angle X-ray scattering
F-MDist: Fitch Margoliash distance
RAxML: randomized accelerated maximum likelihood
PhyML: phylogeny maximum likelihood
HFF: human foreskin fibroblast.
