# Supplementary material for: Functional and phylogenetic evidence of a bacterial origin for the first enzyme in sphingolipid biosynthesis in a phylum of eukaryotic protozoan parasites
Source: J Biol Chem. 2017 Jun 2;292(29):12208–19. doi: 10.1074/jbc.M117.792374 (PMC5519370; doi:10.1074/jbc.M117.792374)
Supplement: Supplemental Data [file supp_292_29_12208__index.html]

Functional and phylogenetic evidence of a bacterial origin for the first enzyme in sphingolipid biosynthesis in a phylum of eukaryotic protozoan parasites — Functional and phylogenetic evidence of a bacterial origin for the first enzyme in sphingolipid biosynthesis in a phylum of eukaryotic protozoan parasites — The apicomplexan serine palmitoyltransferase — Supplemental Data 

# Functional and phylogenetic evidence of a bacterial origin for the first enzyme in sphingolipid biosynthesis in a phylum of eukaryotic protozoan parasites

## Supplemental Data

- Supplemental 1 (.gif, 314 KB) - A SDS-PAGE analyses of 4 TgSPT1 N-terminal deletions of 143, 158, 176 and 180 amino acids (???????????143, ???????????158, ???????????176 and ???????????180) following overexpression in E. coli and isolation as described. B Functional analyses of proteins shown in A. ???????????143, ???????????158, ???????????176 and ???????????180 are reactions with equivalent quantities of protein showing incorporation of 14C serine into lipid species indicated; ??????????????????ve is the negative control with no protein added. Lipids by separated by high performance thin-layer chromatography. O is origin; F is front; KDS denotes 3-ketodihydrosphinganine migrating with the same Rf value previously reported under these conditions (21).
- Supplemental 2 (.gif, 563 KB) - The scatterplot shows no significant correlation between the localisation of native TgSPT1 (antiSPT-AF594, red) and episomally expressed cytosolic GFP (CytoGFP, green).
- Supplemental 3 (.txt, 9 KB) - CLUSTALW multiple sequence alignment in PHYLIP format used as input into all phylogenetic platforms utilised. In this format the number of sequences (34) and the length of alignment (184) are shown top left. The protein sequences are annotated as for Figure 7 in the first block.
