# Supplementary figures and images for: Functional and phylogenetic evidence of a bacterial origin for the first enzyme in sphingolipid biosynthesis in a phylum of eukaryotic protozoan parasites
Source: J Biol Chem. 2017 Jun 2;292(29):12208–19. doi: 10.1074/jbc.M117.792374 (PMC5519370; doi:10.1074/jbc.M117.792374)

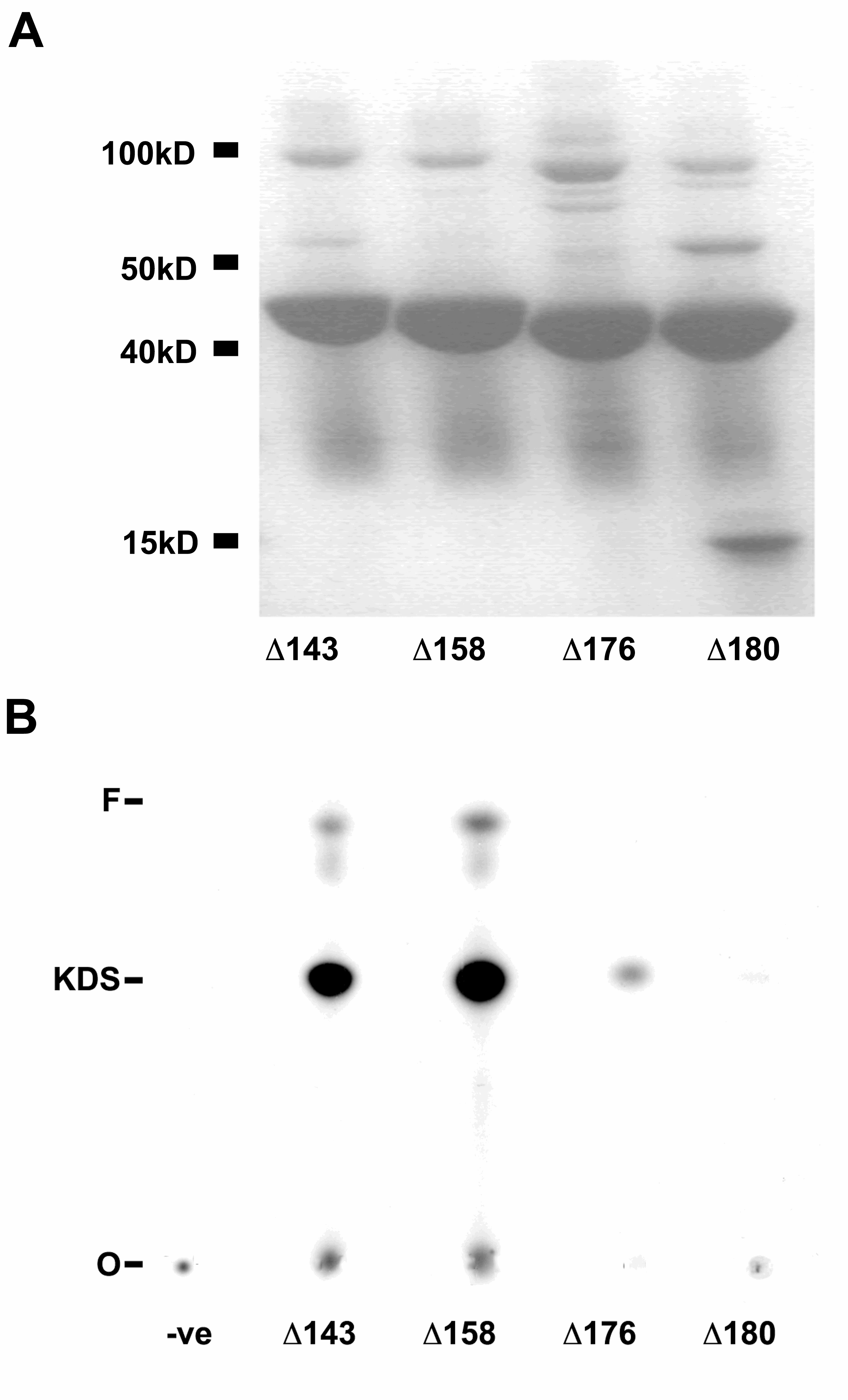

Supplement: Supplemental Data [file 10.1074_M117.792374_jbc.M117.792374-1.gif]

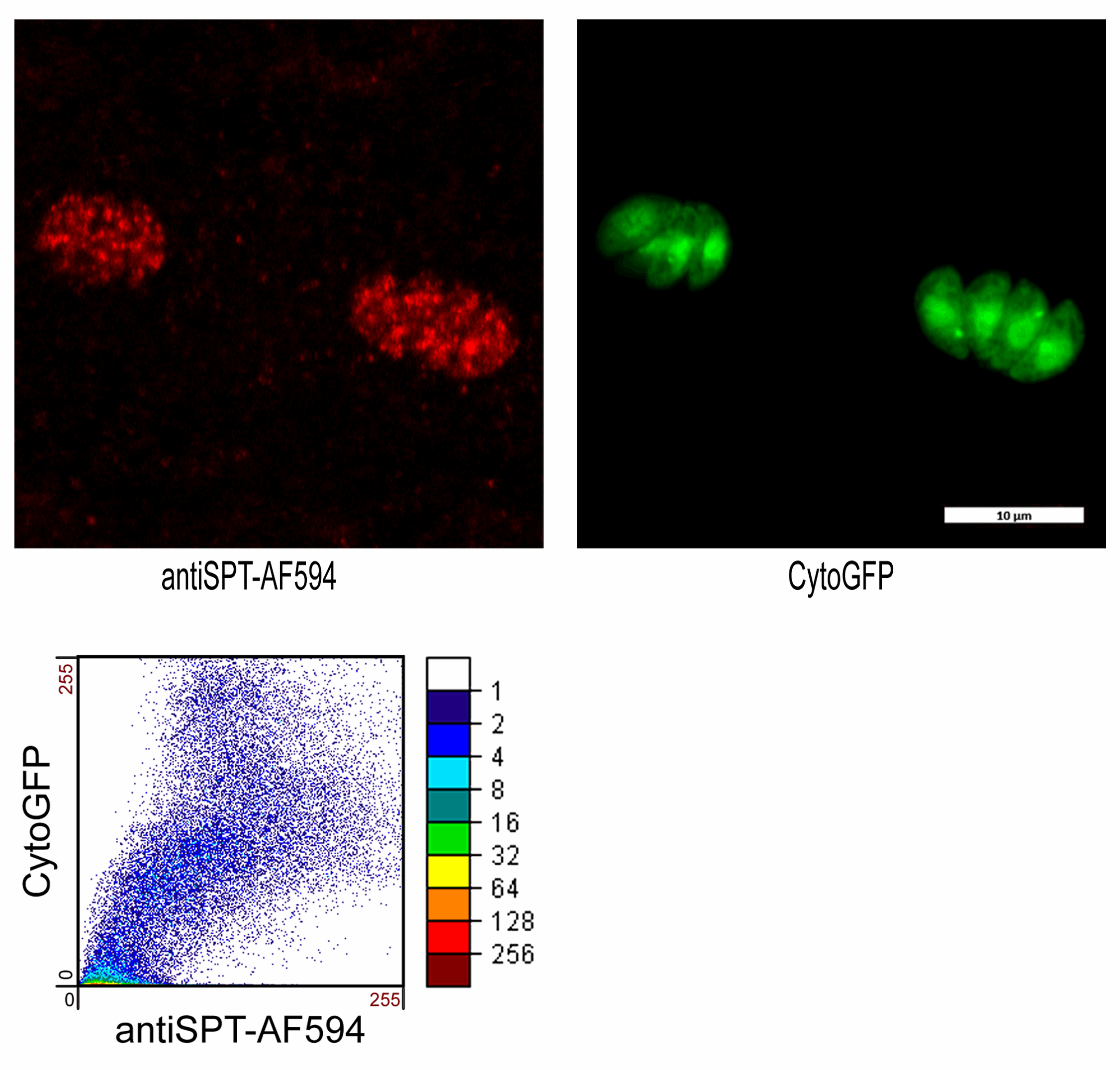

Supplement: Supplemental Data [file 10.1074_M117.792374_jbc.M117.792374-2.gif]
